# Observation of terahertz coherent edge radiation amplified by infrared free-electron laser oscillations

**DOI:** 10.1038/s41598-021-82898-7

**Published:** 2021-02-09

**Authors:** Norihiro Sei, Takeshi Sakai, Yasushi Hayakawa, Yoske Sumitomo, Kyoko Nogami, Toshinari Tanaka, Ken Hayakawa

**Affiliations:** 1 Research Institute for Measurement and Analytical Instrumentation, National Institute of Advanced Industrial Science and Technology, 1-1-1 Umezono, Tsukuba, Ibaraki 305-8568 Japan; 2 Laboratory for Electron Beam Research and Application, Nihon University, 7-24-1 Narashinodai, Funabashi, 274-8501 Japan

**Keywords:** Terahertz optics, Optical physics

## Abstract

A coupling device, which can extract coherent edge radiation (CER) from an optical cavity for a free-electron laser (FEL) without damaging the FEL due to diffraction loss, was developed at Nihon University. We successfully observed the CER beam with a power of 1 mW or more in the terahertz range during FEL oscillation. It is revealed that the CER power changed with the detuning of the optical cavity and the dependence of the CER power on the detuning length differs from that of the FEL power. The measured CER spectra indicate that the longitudinal electron distribution in a bunch is modulated by the FEL oscillation with a period corresponding to the FEL slippage length. We herein report the characteristics of the CER with FEL oscillation in detail. These results demonstrate that the CER is excellent tool to reveal the overall effect of FEL interaction on electron distribution in a bunch.

## Introduction

Free-electron lasers (FELs) have been developed in a broad wavelength range from millimeter waves to X rays. They have been used in various fields, such as basic science and industrial applications^1,2^. To realize FEL oscillations, in addition to techniques for forming an electron bunch with high electron density, techniques for observing the characteristics of the electron bunch are being developed. In particular, the electron distribution in the longitudinal direction not only contributes to the FEL gain, but also affects the FEL characteristics, such as the pulse width and line width^3–5^. A technique for observing the bunch length during FEL oscillations is desired to reveal the FEL interaction and to control lasing stably. For measuring longitudinal electron distributions, excellent techniques such as radio-frequency (RF) deflectors have been developed in recent years^6,7^. However, it is still difficult to nondestructively monitor the electron bunch immediately after an FEL interaction.

Therefore, we observed the coherent edge radiation (CER) emitted in an FEL straight section and measured the bunch shape of the electron beam immediately after an FEL interaction at the infrared FEL facility of the Laboratory for Electron Beam Research and Application (LEBRA) in Nihon University^8^. Edge radiation is generated by a relativistic charged particle when it passes through the region of a rapid change in magnetic field at the edges of the bending magnets. Because the edge radiation has an annular shape distribution characterized by an asymmetric first-order Laguerre-Gaussian mode^9,10^, the CER can be extracted from the optical cavity without a diffraction loss from the FEL beam. Moreover, the intensity of the edge radiation is much higher than that of synchrotron radiation in the terahertz (THz) range, where the edge radiation becomes coherent for linac electron beams^9,11^. Therefore, CER is easier to observe than coherent synchrotron radiation. We installed a hollow concave mirror in front of the downstream mirror of an optical cavity at the LEBRA and successfully observed the CER during infrared FEL oscillations on a macroscopic time scale. We discovered that the measured CER power depended on the detuning length of the optical cavity. Herein, the experimental results for the change in the CER caused by FEL oscillations are reported for the first time.

## Results

### Calculated characteristics of CER beam at LEBRA

**Figure 1:**
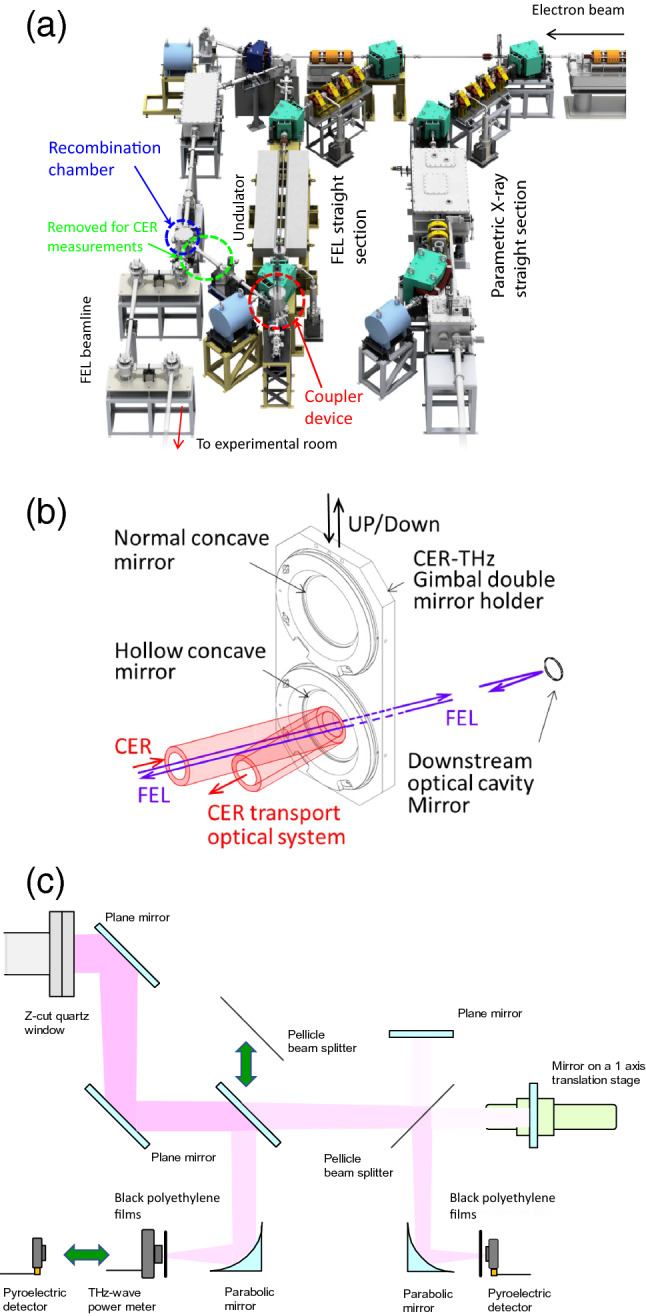
Experimental setup and observation system for CER beam at LEBRA. (**a**) schematic layout of two light-source straight sections and FEL beamline. (**b**) switchable normal concave and hollow concave mirrors installed in the coupler device. (**c**) schematic layout of observation system for CER beam installed between the coupler device and recombination chamber. A plane mirror and a THz-wave power meter were used to the measure of the CER power, whereas a pellicle beam splitter and a pyroelectric detector were used to measure the CER spectra. (**a**) is permitted for publication under a CC BY open access license by the copyright holder Mr. Mamoru Horiuchi.

The LEBRA has an S-band linac with an electron energy range of 40–100 MeV. As shown in Fig. 1a, the electron beam from the linac is supplied to two straight sections and used in each straight section for wavelength-tunable monochromatic light sources, namely an infrared FEL and a parametric X ray^12,13^. As described in the Method section, a coupler device that can extract the CER beam generated at the downstream bending magnet without losing the FEL beam was installed in the optical cavity^8^. This coupler device can insert a normal concave mirror or a concave mirror with a 25-mm diameter hole on the optical axis at a position 0.82 m from the CER generation point, as shown in Fig. 1b.

**Figure 2:**
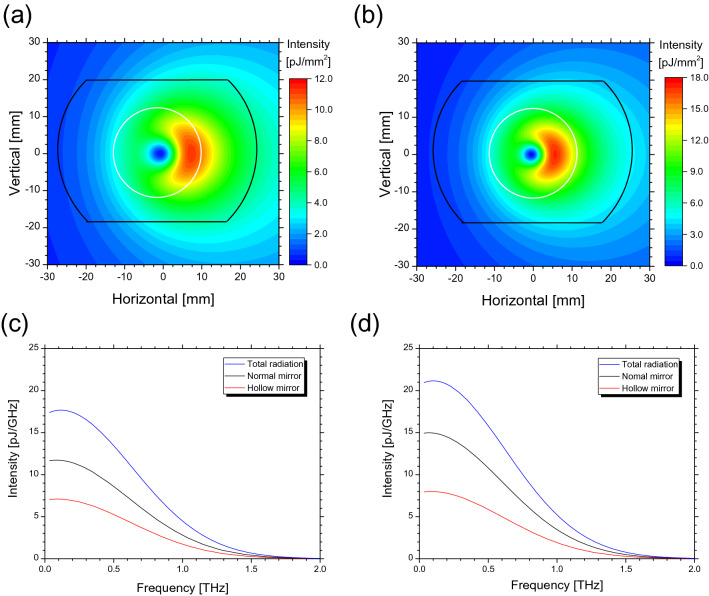
Calculated characteristics of CER beam generated by an electron bunch with a charge of 27.6 pC and an RMS bunch length of 0.06 mm. Spatial distributions of CER beam for electron energies of 58 meV (**a**) and 75 MeV (**b**). White and black solid lines express boundaries of hole of hollow concave mirror and area in which CER is directly irradiated on the mirror surface, respectively. Calculated spectra of CER beam for electron energies of 58 MeV (**c**) and 75 MeV (**d**). Blue, black, and red solid lines are spectra of CER beam irradiated at all solid angles on the normal and hollow concave mirrors, respectively.

Figure 2 shows the spatial distributions of CER beams calculated using the CER theory for electron energies of (a) 58 MeV and (b) 75 MeV on a concave mirror^9,14^. In these calculations, the root-mean-squared (RMS) bunch length and charge of the electron bunch were assumed to be 0.060 mm and 27.6 pC, respectively. A boundary, inside which the CER was directly irradiated without reflection by the inner vacuum chamber, and a coupling hole are illustrated as black and white lines in this figure, respectively. It was discovered that the spatial distribution of the CER beam depended on the electron energy and the CER profile was more compact as the electron energy increased. The spatial distribution of the CER beam depended on the frequency as well. Figure 2c and d show the calculated spectra of the CER beam that directly irradiated the normal and hollow concave mirrors, respectively, in addition to the spectrum of the CER integrated at all solid angles. The CER beam reflected on either of the concave mirror can be transported to the atmosphere through the z-cut quartz window without colliding with the vacuum chambers. It is measured by the observation system as shown in Fig. 1c. Subsequently, we observed the transported CER spectra accounted for the effect of the window transmittance and the atmosphere absorption^15, 16^. It was observed that all the spectra shown in Fig. 2c and d exhibited similar frequency dependences. The normal and hollow concave mirrors can extract approximately two-thirds of the total CER power and approximately one-third of the total CER power, respectively.

### Measured characteristics of the CER beam in LEBRA

**Figure 3:**
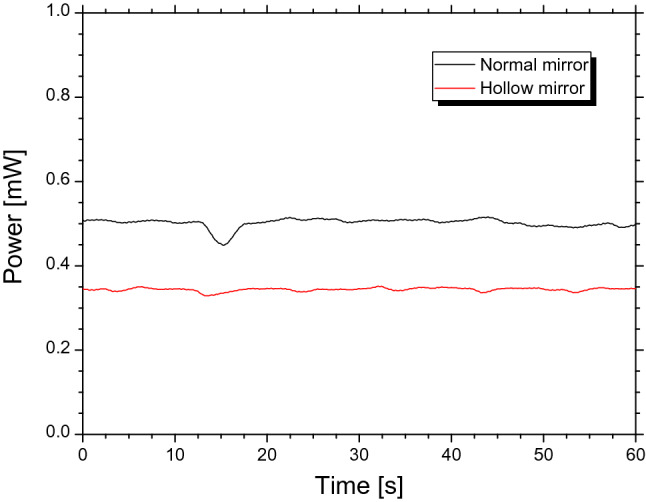
Measured power of CER beam. Black and red lines represent CER power measured with normal and hollow concave mirrors, respectively.

As the electron energy is higher in the LEBRA, the electron bunch is shorter, and the electron distribution in the bunch becomes more complicated. In addition, the electron bunch shape tends to be distorted from the ideal Gaussian distribution when the charge in the bunch increases^17,18^. It is relatively difficult to use the complex electron distribution to evaluate the characteristics of an electron bunch via observations of coherent radiation. Therefore, we observed the CER at an electron energy of 58 MeV in the full bunch mode, where electron bunches filled whole S-band RF-buckets^19^. The macropulse duration of the electron beam was 18.1 μs, and the averaged charge of the electron bunch was 27.6 pC. First, the mirrors in the coupler device were removed from the FEL beam in the optical cavity, and the FEL system was adjusted to maximize the FEL power. The measured power of the FEL was 1.5 mW and its macropulse duration was 6.4 μs. The *K* value was set to 1.87, and the wavelength of the FEL was 5.4 μm. Next, the hollow concave mirror was inserted in the FEL beam, and the THz-wave power on the FEL oscillations was measured. THz waves with a power of 0.35 mW were observed, as shown in Fig. 3. The FEL power was 1.5 mW, which was the same as before the hollow concave mirror was inserted. Therefore, it was confirmed that the FEL oscillation was not disturbed by the hollow concave mirror. When the hollow concave mirror was switched to the normal concave mirror, the power of the THz waves increased to 0.50 mW.

**Figure 4:**
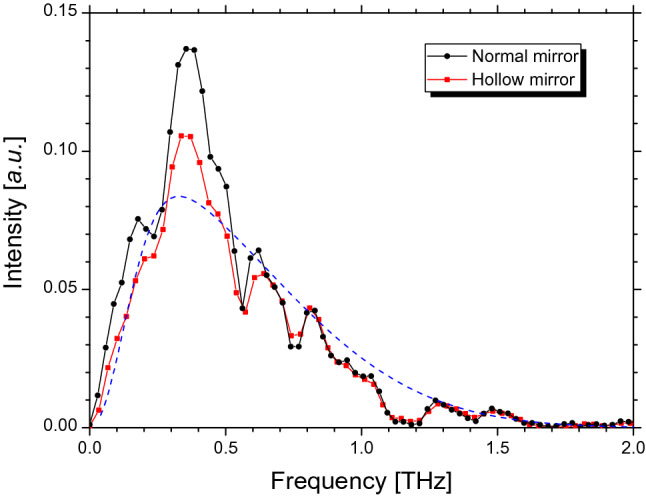
Measured spectra of CER beam at the electron energy of 58 MeV. Black and red lines represent CER power measured with normal and hollow concave mirrors, respectively. Dotted blue line represents fitting curve for spectrum measured using hollow concave mirror.

We measured the spectra of the THz waves reflected by two types of concave mirrors using a Michelson interferometer. As shown in Fig. 4, the two measured spectra had similar frequency structures. Because the spectral measurements were performed in the atmosphere, dips appeared owing to the absorption of water vapor at the frequencies of 0.56, 0.75, 0.99, 1.1–1.2, and 1.4 THz in the measured spectra^16^. Moreover, the measured spectra decreased rapidly below 0.35 THz. This decrease was caused by the diffraction loss of the transport optical system of the CER beam. Although all optical elements in the transport optical system caused the diffraction loss, it is typically sufficient to consider a diffraction loss where the ratio of the diameter of the optical element to the CER beam size is the smallest^14^. The observed spectrum *O*(*ω*) expressed as the following approximate expression using the CER spectrum *U*(ω):

1 $$O\left( \omega \right) \cong \left[ {1 - {\text{e}}^{{ - {{\omega^{2} } \mathord{\left/ {\vphantom {{\omega^{2} } {\omega_{c}^{2} }}} \right. \kern-\nulldelimiterspace} {\omega_{c}^{2} }}}} } \right]^{a} T\left( \omega \right)U\left( \omega \right),$$

where *T*(*ω*) is the transmittance spectrum from the light source to the detector, and *ω*_*c*_ is the minimum cutoff angular frequency in the optical system^20^. Although the exponent *a* is 2 for the axisymmetric light source and optical elements, it is 1 for the CER beam because of the axial asymmetry. The cutoff frequency evaluated from the frequency at which the observed CER spectrum indicated a maximum was approximately 0.18 THz. Considering the diffraction loss and *T*(*ω*), the electron distribution in the bunch can be estimated from the measured spectra at frequencies higher than the cutoff frequency. In our experiments, *T*(*ω*) was expressed as a product of the transmittances of the z-cut quartz window^15^, black polyethylene films attached in front of the detector^21,22^, and the atmosphere^16^. Because the electron energy was relatively low, the longitudinal electron distribution in the bunch was assumed to be Gaussian distribution. The dotted curves shown in Fig. 4 were obtained by applying the least-squares approximation to the spectrum measured using the hollow concave mirror at the frequencies of 0.37, 0.47, 0.64, 0.84, 0.94, 1.04, 1.28, and 1.48 THz^14^. The measured data were not affected by the water vapor absorption at these frequencies. The fitting curve shows that the RMS bunch length was 0.06 mm. Using Eq. (1), the CER powers in the measurement system at the LEBRA were calculated to be 0.30 mW for the hollow concave mirror and 0.49 mW for the normal concave mirror. The calculated CER-beam powers assuming a Gaussian electron distribution were approximately consistent with the measured powers. The experimental results indicate that the observed THz waves were the CER beam. For the first time, we successfully observed the CER generated by the electron beam immediately after an FEL oscillation.

### CER power with optical cavity detuning

**Figure 5:**
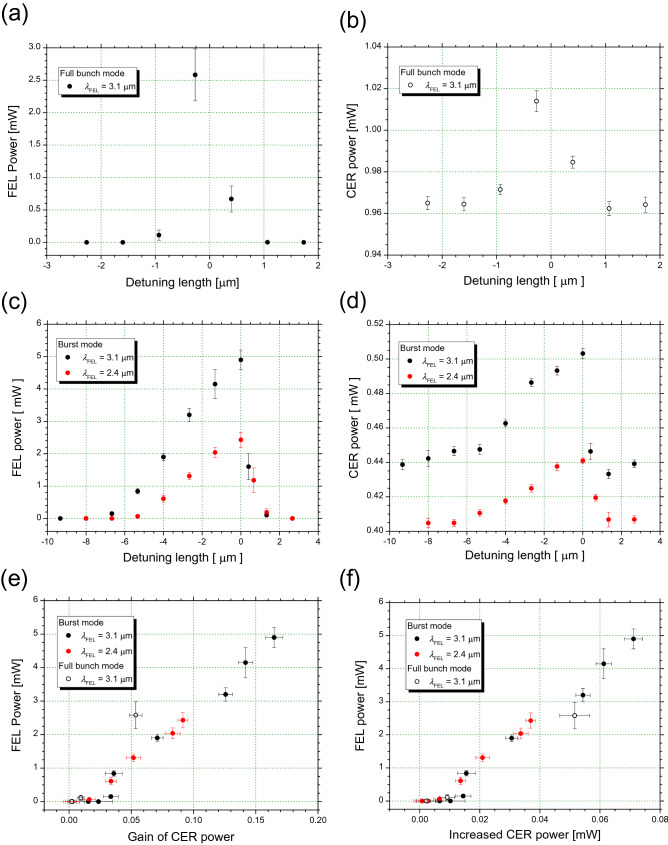
Relationship between CER and FEL powers with optical cavity detuning. Dependence of FEL power (**a**) and CER power (**b**) on detuning length in full bunch mode. Dependence of FEL power (**c**) and CER power (**d**) on detuning length in burst mode. Black and red circles represent data for FEL wavelengths of 3.1 and 2.4 μm, respectively. Medians of FEL detuning curve were − 1.70 and − 1.16 μm for FEL wavelengths of 3.1 and 2.4 μm, respectively. Medians of CER detuning curve were − 2.15 and − 1.39 μm for FEL wavelengths of 3.1 and 2.4 μm, respectively. Data of CER power and error bars in (**b**) and (**d**) represent average value and dispersion for 30 s, respectively. Dependence of gain of CER power (**e**) and net increment of CER power (**f**) on FEL power. Black and red symbols represent data for FEL wavelengths of 3.1 and 2.4 μm, respectively. Solid and open circle symbols represent data in burst and full bunch modes, respectively.

For the hollow concave mirror, the measured power of the THz waves was slightly higher than the calculated power of the CER beam. It was observed that the CER power tended to decrease (~ 0.01 mW) when the FEL oscillation was stopped by adjusting the optical cavity length. Therefore, to clarify the correlation between the CER and FEL powers, experiments were conducted under an electron energy of 75 MeV. In the linac, generally, the RMS bunch length decreases as the acceleration gradient increases^23^. Although the high electron density distorts the bunch shape from the ideal Gaussian distribution, the power of the coherent radiation is expected to increase with the electron energy. We generated a CER beam using an electron beam with a macropulse duration of 17.6 μs and a bunch charge of 29.4 pC in the full-bunch operation. The CER power reflected by the hollow concave mirror, which was measured using a THz power meter, was 0.96 mW without FEL oscillations. By increasing the optical cavity length by 13 μm, the FEL oscillated at a wavelength of 3.1 μm. The macropulse duration and power of the FEL were 4 μs and 2.6 mW, respectively. The value measured from the THz-wave power meter increased to 1.04 mW. Because the FEL and its higher harmonics were absorbed by the black polyethylene films^21,22^, it can be concluded that the light power in the THz range increased owing to the FEL oscillation. Figure 5a shows the relationship between the FEL power and optical cavity detuning, and Fig. 5b shows the relationship between the THz-wave power and optical cavity detuning. As shown in these figures, the THz-wave power indicated a maximum at approximately zero detuning length, similar to the FEL power^24^. The net gain of the THz waves increased up to 5%.

Because the power meter had a long time constant, we could not contradict that the unexpected temperature elevation of the concave mirror caused by the FEL oscillation affected the increase in the THz-wave power. Therefore, we investigated the relationship between the THz-wave power and the detuning length of the FEL optical cavity in the burst mode^25^, where the power of the FEL micropulse was higher than that in the full bunch mode. The electron energy was set to 75 MeV, and the macropulse duration in the electron beam was 17.4 μs. Although the charge of the electron bunch was 360 pC, only approximately one-third of the charge contributed to the FEL oscillation by bunch compression^26^. The *K* value was set to 1.87, and the FEL oscillated at a wavelength of 3.1 μm. The duration of the FEL macropulse was as long as 13 μs, and the maximum FEL power was 5.0 mW. Figure 5c and d show the dependences of the FEL power and THz-wave power on the optical cavity detuning in the burst mode, respectively. Although the CER power without FEL oscillations was as low as 0.43 mW, the maximum gain of the THz waves was 16% or more. When the detuning length was negative, the THz-wave power became larger before FEL oscillations were detectable. Meanwhile, when the detuning length was positive, the gain of the THz-wave power decayed faster than the FEL power. It was found that the median of the THz-wave amplification was located in the negative direction of the detuning length compare to that of the FEL power. We changed the *K* value of the undulator to 1.53 and adjusted the FEL wavelength to 2.4 μm. Subsequently, a similar relationship between the THz-wave power and detuning length was observed, as shown in Fig. 5d. The correlation between the FEL power and THz-wave amplification is summarized in Fig. 5e and f. It was revealed that the increase in the power and gains of the THz waves with respect to the FEL power differed depending on the electron beam mode. These experimental results indicate that the increase in the THz-wave power was not caused by the temperature elevation of the concave mirror owing to the FEL oscillation. Because radiation with high power did not exist in the THz range except for the CER, it can be concluded that the increase in the THz waves was due to the increase in the CER beam. The difference in the correlations between the FEL power and CER amplification due to the electron bunch mode suggests that the CER amplification depends on the characteristics of the FEL micropulse.

### Observation of CER spectrum on FEL oscillations

**Figure 6:**
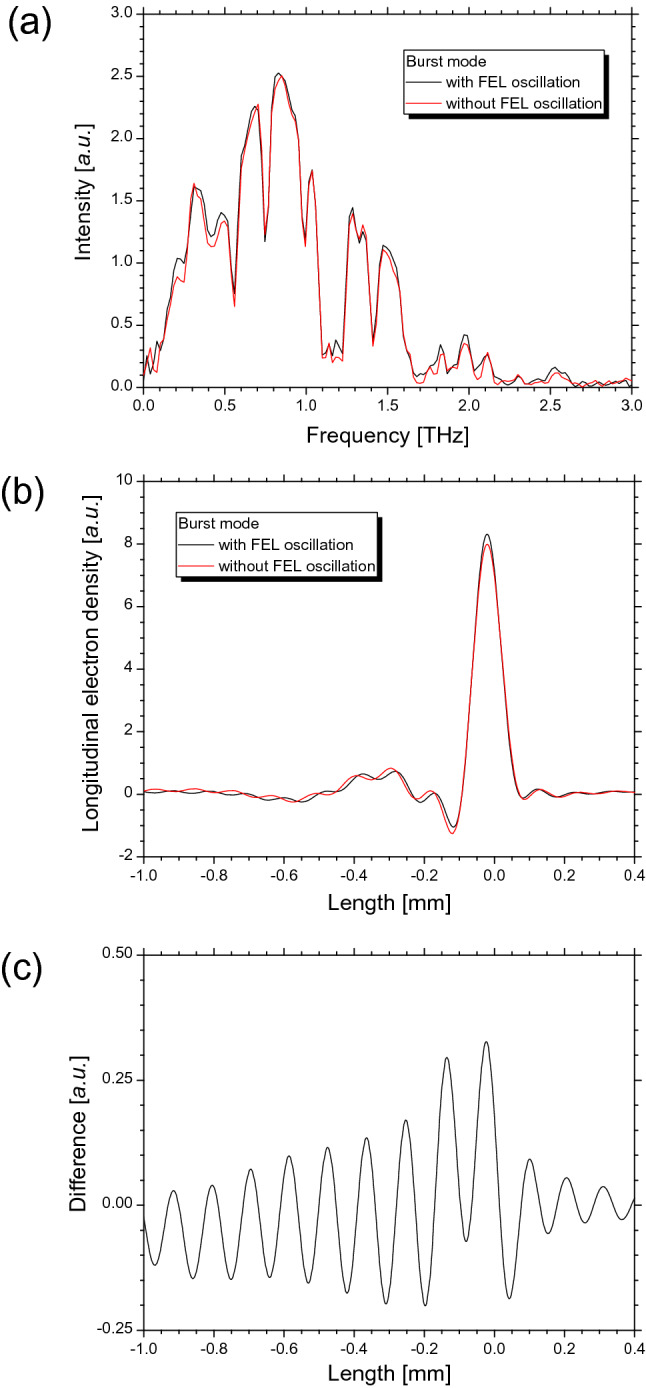
Observation of changes in CER spectrum and electron-bunch shape due to FEL oscillations at the electron energy of 75 MeV. In (**a**), spectrum of CER beam with (black line) and without (red line) FEL oscillations in burst mode. In (**b**), longitudinal electron-bunch shapes calculated by measured CER spectra with (black line) and without (red line) FEL oscillations. Electron densities were normalized such that the electron numbers were equal in both electron-bunch shapes. In (**c**), difference in normalized electron density with FEL oscillations minus normalized electron density without FEL oscillations in (**b**).

To investigate the frequency components of the CER beam increased by FEL oscillations, we measured the CER spectrum using a hollow concave mirror with and without FEL oscillations. To increase the gain of the CER power, the electron beam was operated at an electron energy of 75 MeV in the burst mode. Approximately 30 min was required to measure the CER spectrum using the Michelson interferometer. The FEL at a wavelength of 3.1 μm oscillated to maintain a power of 1 mW or more without adjusting the electron beam during the spectral measurement. Furthermore, we measured the CER spectrum without FEL oscillations by shortening the optical cavity length by 38 μm. Figure 6a shows the measured CER spectra with and without FEL oscillations. The CER was amplified by the FEL oscillation at frequencies of approximately 2.0 THz and in a frequency region below 0.6 THz. By considering the diffraction loss of the transport optical system and the transmittances of the z-cut quartz window, water vapor, and polyethylene films, the form factor was calculated based on the measured CER spectrum^18^. Figure 6b shows the longitudinal electron-bunch shapes estimated from the form factor calculated using the Kramers–Kronig relation^27,28^. Full widths at half maxima of the calculated spectra with and without the FEL oscillations were approximately 0.085 and 0.087 mm, respectively. The two calculated electron-bunch shapes were matched the peak positions. The electron density in the bunch increased near the peak owing to FEL oscillations, thereby amplifying the CER power. As shown in Fig. 6c, where the difference between the electron distributions with and without FEL oscillations was calculated, the electron density was modulated with a period of 0.12 mm near the peak. This period agreed relatively with the FEL slippage length of 0.11 mm^29,30^. Our experimental results suggest that the electron distribution in the bunch would be affected by the length of the FEL interaction.

## Conclusion and discussion

We developed a system for observing the CER generated by an electron beam immediately after an FEL interaction at the LEBRA. By arranging a hollow mirror in the optical cavity, a CER beam with a total power of 30% or more can be extracted during the FEL oscillation without causing a diffraction loss in the FEL. The bunch length with FEL oscillations was evaluated by measuring the spectrum of the CER beam, which was a high-power THz wave. Furthermore, it was discovered that the CER power varied with the detuning length of the optical cavity. Although the detuning curve of the CER amplification was similar to that of the FEL power, the median of the CER power shifted in the negative direction of the detuning length compare to that of the FEL power. By measuring the CER spectrum with FEL oscillations, it was clear that the electron density in the bunch increased near the peak owing to the FEL oscillations, thereby amplifying the CER power. Moreover, the longitudinal electron density was modulated with a period corresponding to the slippage length of the FEL. In these experiments, we did not quantitatively evaluate the correlation between the FEL power and electron bunch modulation. Nevertheless, we could conduct the first study regarding the reaction of FEL oscillations to the electron bunch.

Although the THz detectors used in this study were suitable for observing the macro-temporal properties of the CER, they could not measure the micropulse of the CER because of the long time constant. To observe the longitudinal electron distribution for each micropulse, the CER spectra should be measured using multiple narrow-band diode detectors with high temporal resolutions, as demonstrated by the corresponding author at the Kyoto University Free Electron Laser^14^. We plan to measure the evolution of the CER spectra in a macropulse with narrow-band diode detectors while monitoring the FEL oscillation. Real-time measurements of the CER spectra will reveal the effect of FEL oscillations on the electron distribution in the bunch in more detail. Moreover, coherent radiation is attractive as a high-power THz-wave source^31^. The THz-wave CER beam amplified by the FEL oscillation can be transported to the experimental room simultaneously with the infrared FEL beam at the LEBRA^32^. Using a composite light source composed of a THz CER and an infrared FEL is expected to birth cutting-edge applications.

## Method

### Infrared FEL device at LEBRA

The S-band linac at the LEBRA comprises a 100 keV DC electron gun with a high-speed grid pulsar, prebuncher, buncher, and three 4-m-long traveling wave accelerator tubes^25^. In the FEL experiments, the electron energy was accelerated from 55 to 100 MeV by the linac, and the macropulse of the electron beam was operated at a frequency of 2 Hz. The macropulse duration determined by the flat-top pulse width of the 20 MW klystron output power was approximately 20 μs. As shown in Fig. 1a, the accelerated electron beam was guided to an FEL straight section by two 45° bending magnets. The electron micropulse was compressed when it passed through the two bending magnets, and the bunch length of the electron beam was less than 0.15 mm^33^. The insertion device is a 2.4-m planar undulator with the period number of 50, and the maximum *K* value was 1.87 in the FEL experiments. However, because the magnets on the upstream side of the undulator were demagnetized by the electron beam, the effective period number of the undulator with a magnetic field error of less than 3% was 35 in these experiments. The electron beam in the undulator was removed from the FEL straight section using a 45° bending magnet, and it lost its energy in a beam dump. Mirror chambers, each containing a metal mirror with a radius of curvature of 4 m and a diameter of 25.4 mm, were set at the ends of the FEL straight section. The mirrors were placed 6.72 m apart. Fundamental FELs oscillated in the wavelength range of 1–6 μm. The FEL that passed through a 0.4-mm-diameter hole in the upstream cavity mirror was formed into a parallel beam. The FEL beam was reflected by 10 vapor-deposited aluminum mirrors and transported from the optical cavity to the next experimental room. The transported FEL was extracted into the atmosphere through a calcium fluoride window and measured using a power meter (PE25BB-S, Ophir Optronics Solutions Ltd.). It is applied for basic research on dentistry and the development of new materials^34,35^. Two macropulse modes of the electron beam, i.e., the full bunch mode and the burst mode, are operated at the LEBRA^25^. In the full bunch mode, the electron bunch is accelerated in each period of the 2856 MHz accelerating RF field, and the maximum charge of the electron bunch is approximately 30 pC. In the burst mode, the high-speed grid pulsar with a pulse width of 600 ps is operated at intervals of 22.4 ns. Typically, two electron bunches that have a charge of exceeding 300 pC per bunch are accelerated. The average current of the electron beam in the burst mode is lower than that in the full bunch mode. However, the width of the FEL macropulse in the burst mode is longer than that in the full bunch mode, and the FEL peak power in the burst mode is higher than that in the full bunch mode.

### Coupling device to extract CER from FEL resonator

To observe the effect of the FEL interaction on the electron bunch in the FEL straight section, we developed a coupler device that can extract the CER beam generated at the downstream bending magnet without losing the FEL beam in the optical cavity^8^. As shown in Fig. 1b, this coupler device can insert a normal concave mirror or a concave mirror with a 25-mm diameter hole on the optical axis in the center at a position 0.82 m away from the CER generation point. Each mirror was made of an aluminum substrate with gold deposited on the surface, and the radius of the curvature was 1.2 m. Although the diameter of the concave mirror was 70 mm, the joint between the vacuum chambers of the coupler device and the downstream bending magnet was narrow. The effective diameter irradiated by the CER onto the mirror without reflection by the inner vacuum chamber was 53.7 mm. Furthermore, the area in which the CER was directly irradiated was limited to the vertical direction by the vacuum chamber of the downstream bending magnet. The effective length on the mirror was 38.6 mm in the vertical direction. The CER beam that was deflected 135° by the concave mirror was transported to the recombination chamber installed in the FEL beamline. The inner diameter of the beam pipe connecting the recombination chamber and coupler device exceeded 60 mm. A sapphire substrate deposited with indium tin oxide with a diameter of 76 mm can be installed in the recombination chamber. The THz-wave CER beam  reflected by the sapphire substrate can be transported to the experimental room coaxially with the near-infrared FEL passing through the sapphire substrate. Considering the diffraction of the THz-wave CER, part of the beam pipe was removed in this study. A z-cut quartz window with a diameter of 100 mm was installed at a position 1.5 m from the concave mirror, and the characteristics of the CER beam were measured in the atmosphere.

### Observation system for CER beam

The CER beam extracted into the atmosphere was focused using a parabolic mirror of diameter 76 mm and focal length 153 mm. The power of the CER beam was measured using a THz-wave power meter (3A-P-THz, Ophir Optronics Solutions Ltd.) with a time constant of 0.8 s. Four black polyethylene films with a thickness of 25 μm were placed in front of the power meter. They blocked lights from mid-infrared to visible ranges, and the transmittance was less than 0.1% in a wavelength range of 6 μm or less^21,22^. Meanwhile, the transmittance exceeded 85% in the frequency range of 2 THz or less. When the spectra of the CER beam were measured, the flat mirror that transported the CER beam to the THz-wave power meter was replaced with a beam splitter, which was a nitrocellulose pellicle of a thickness 2 μm and diameter 108 mm. This pellicle was coated with a nickel-based alloy, and the transmittance and reflectance in the THz region were approximately 60% and 10%, respectively^14^. The CER beam transmitted through the pellicle was injected into a Michelson interferometer with another pellicle as a beam splitter. The interfering CER beam was focused using a parabolic mirror of diameter 76 mm and focal length 101 mm. The intensity of the interfering light was measured using a pyroelectric detector (THz10, Sensor-und Lasertechnik Inc.) with a time constant of 0.1 ms. Four black polyethylene films with a thickness of 25 μm were placed in front of the pyroelectric detector to block the lights except THz waves. Because the CER beam from the pellicle beam splitter installed upstream of the Michelson interferometer was used as a reference light, RMS noise of the interferogram was as low as 1%. The outline of the CER observation system is shown in Fig. 1c. The distance that the CER passed through the atmosphere from the vacuum window to the detector was approximately 1.9 m.

### CER theory

The spectrum of the CER is essential for measuring the bunch shape. The angular spectral energy distribution of the CER at angular frequency *ω* is expressed as

2 $$\frac{dU}{{d\omega d\Omega }} = N\left( {N - 1} \right)f\left( \omega \right)\frac{{dU_{ER} }}{d\omega d\Omega } \cong N^{2} f\left( \omega \right)\frac{{dU_{ER} }}{d\omega d\Omega },$$

where *N* is the number of electrons in a bunch and *Ω* is the solid angle^9^. The angular spectral energy distribution of the edge radiation per electron, *dU*_*ER*_/*dωdΩ*, is expressed as the horizontal observation angle *Φ* and vertical observation angle *Θ* as follows:

3 $$\frac{{dU_{ER} }}{d\omega d\Omega } = \frac{{e^{2} \gamma^{2} }}{{4\pi^{3} \varepsilon_{0} c}}\left[ {A + B + C + D - E} \right],$$

4 $$A = \frac{{\gamma^{2} \Phi^{2} }}{{\left( {1 + \gamma^{2} \Theta^{2} + \gamma^{2} \Phi^{2} } \right)^{2} }},$$

5 $$B = \frac{\gamma \Phi }{{1 + \gamma^{2} \Theta^{2} + \gamma^{2} \Phi^{2} }}\frac{2\pi }{{\sqrt[3]{3}\Gamma \left( {{1 \mathord{\left/ {\vphantom {1 3}} \right. \kern-\nulldelimiterspace} 3}} \right)}}\sqrt[3]{{\frac{3\omega }{{4\omega_{c} }}}},$$

6 $$C = \frac{\pi }{{\sqrt[3]{3}\Gamma \left( {{1 \mathord{\left/ {\vphantom {1 3}} \right. \kern-\nulldelimiterspace} 3}} \right)}}\left( {1 + \frac{1}{\sqrt 3 }} \right)\sqrt[3]{{\left( {\frac{3\omega }{{4\omega_{c} }}} \right)^{2} }},$$

7 $$D = \frac{{\gamma^{2} \Theta^{2} }}{{\left( {1 + \gamma^{2} \Theta^{2} + \gamma^{2} \Phi^{2} } \right)^{2} }},$$

8 $$E = \frac{{\gamma^{2} \Theta^{2} }}{{1 + \gamma^{2} \Theta^{2} + \gamma^{2} \Phi^{2} }}\frac{2\pi }{{\sqrt[6]{{3^{7} }}\Gamma \left( {{1 \mathord{\left/ {\vphantom {1 3}} \right. \kern-\nulldelimiterspace} 3}} \right)}}\sqrt[3]{{\left( {\frac{3\omega }{{4\omega_{c} }}} \right)^{2} }},$$

where *e*, *c*, *ε*_0_, *γ*, and *Γ* are the charge of the electron, speed of light, permittivity of free space, Lorentz factor, and Gamma function, respectively. The radius of the curvature of the downstream bending magnet in the FEL straight section was 0.55 m, and the angular critical frequency of the synchrotron radiation, *ω*_*c*_, was 137 THz at an electron energy of 75 MeV. Because the power of the coherent synchrotron radiation was much lower than that of the CER, it was negligible in the measurement of the CER power. The form factor *f*(*ω*) was calculated using the Fourier transform of the normalized longitudinal electron distribution *S*(*z*) within the bunch^36, 37^ as follows:

9 $$f\left( \omega \right) = \left| {\int_{ - \infty }^{\infty } {\exp \left( {i\frac{\omega }{c}z} \right)S\left( z \right)dz} } \right|^{2}$$

The bunch shape typically deviates from the ideal Gaussian distribution after a magnetic compression in an arc section. However, it is significant to evaluate the general characteristics of the electron bunch by fitting the bunch shape with a Gaussian distribution. The form factor is expressed as

10 $$f\left( \omega \right) = {\text{e}}^{{ - {{\omega^{2} \sigma_{z}^{2} } \mathord{\left/ {\vphantom {{\omega^{2} \sigma_{z}^{2} } {c^{2} }}} \right. \kern-\nulldelimiterspace} {c^{2} }}}}$$

where *σ*_*z*_ is the RMS bunch length calculated using the least-squares method and applied to the CER spectrum of the specific frequency region. Conversely, the longitudinal electron distribution can be calculated by evaluating the form factor with a measured CER spectrum. The phase information Ψ(*ω*), which is lost when calculating the form factor, can be recovered by applying the Kramers–Kronig relation as follows^27,28^:

11 $$\Psi \left( \omega \right) = \frac{2\omega }{\pi }\int_{0}^{\infty } {\frac{{\ln \left[ {\sqrt {{{f\left( \nu \right)} \mathord{\left/ {\vphantom {{f\left( \nu \right)} {f\left( \omega \right)}}} \right. \kern-\nulldelimiterspace} {f\left( \omega \right)}}} } \right]}}{{\omega^{2} - \nu^{2} }}d\nu } .$$

The lowest frequency at which the form factor of the CER is negligible, *ν*_*M*_, can be selected at the effective upper limit of integration. The longitudinal electron distribution is expressed as

12 $$S\left( z \right) = \frac{2}{c}\int_{0}^{{\nu_{M} }} {\sqrt {f\left( \nu \right)} \cos \left( {\frac{2\pi \nu }{c}z + \Psi \left( \nu \right)} \right)d\nu } .$$
